# High failure rate of the Duraloc Constrained Inlay

**DOI:** 10.3109/17453670903316819

**Published:** 2009-10-01

**Authors:** Gerold Labek, Erich Brabec, Stephan Frischhut, Martin Krismer

**Affiliations:** Department of Orthopaedic Surgery, Medical University InnsbruckInnsbruckAustria

## Abstract

**Background and purpose** After total hip arthroplasty, dislocations are a frequent complication and are difficult to treat in some patients. A great variety of implants and antiluxation mechanisms are used in surgical therapy.

**Methods** 8 patients had 9 Duraloc Constrained Inlays implanted at our clinic between October 2003 and November 2006, for recurrent dislocations. A retrospective follow-up study was carried out.

**Results** All patients suffered a failure of the expanding ring, the metal ring being squeezed out of the polyethylene notch. The mechanism of failure can be explained by impingement due to the implant design. At the time of writing, 3 patients have had to undergo revision surgery.

**Interpretation** The Duraloc Constrained Inlay has shown unacceptably high failure rates.

## Introduction

The Duraloc Constrained Inlay is one of many methods used for revision of recurrent dislocations after total hip arthroplasty. In our department, this inlay was implanted from October 2003 through November 2006 for recurrent dislocations.

After 2 patients had had to be revised because of inlay failure within just a few months in 2007, all patients treated with this inlay at our department were called in for a retrospective follow-up in order to check for further cases of failure in this group of patients, who were considered to be at potentially high risk.

## Patients and methods

The patients were identified with the help of the regional Tyrolean Arthroplasty Register, as well as by means of the in-house operation documentation and the manufacturer's delivery documents. The complete medical histories of the patients, as far as they were associated with arthroplasty treatment, were analyzed in retrospect.

8 patients had been treated with 9 Duraloc Constrained Inlays: in 1 patient a failing Duraloc inlay had been revised by replacing it with the same system (Table). On average, 4.4 (2–10) incidents had occurred before implantation of the inlay. The period between primary and revision surgery averaged 20 (1–72) months.

**Table:** Data on 7 patients treated with the Duraloc Constrained Inlay because of recurrent dislocation

Patient no.	Stem	Cup size	Number of dislocations	Number of operations prior to implantation of the Duraloc Constrained Inlay	Clinical outcome	Revision surgery	Reason for revision
1	Accolade (Stryker)	54	3	2	Ring dislocated	No	
2	CBC (Mathys)	52	2	3	2 revisions due to dislocation on the same hip	Yes	Recurrent dislocation (twice)
3	CBC (Mathys)	54	5	2	Ring dislocated	No	
4	ABG( Stryker)	54	> 6	4	Revision	Yes	Fracture, metallosis
5	CBC (Mathys)	60	> 3	3	Ring dislocated	No	
6	SP II (Link)	54	3	2	Revision	Yes	Aseptic stem loosening
7	CBC (Mathys)	54	2	2	Ring dislocated	No	

Excluding the 2 patients who had been revised (cases no. 2 and 4), the average time interval between implantation and the follow-up examination was 19 (9–47) months. 7 patients (8 hips) had clinical and radiographic follow-up. 1 patient could be reached by phone, but he declined to be examined.

## Results

Implant failure was detected in all patients who had been treated with the Duraloc Constrained Inlay because of recurrent dislocation (Table). Either revision surgery with an exchange of the cup implant had already been carried out (3 patients), or the expanding ring was dislocated (5 patients).

**Figure 1.**
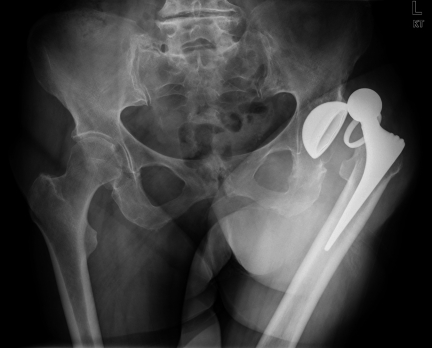
Case no. 2: implant failure and dislocation of the second Duraloc Constrained Inlay.

1 patient (case no. 2) underwent revision surgery due to failure of the antiluxation inlay 3 years after implantation. At revision surgery, the same type of inlay was used again. After 1 more year and another event of dislocation, this inlay was replaced with a Ganz cup and a Mueller antiluxation inlay. He used a brace for 6 weeks after surgery and no further dislocations had occurred a the latest follow-up 2 years after the intervention (Figure 1).

**Figure 2.**
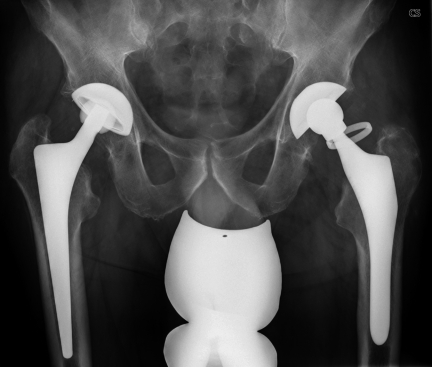
Case no. 4 before revision surgery.

**Figure 3.**
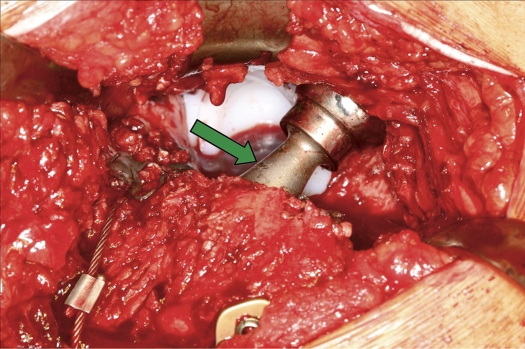
Case no. 4: intraoperatively, with defects at the neck of the stem.

1 patient (case no. 4), after change to the Durolac Constrained Inlay 7 years after primary hip arthroplasty, had to undergo another revision surgery after 10 months due to a periprosthetic fracture in the area of the major trochanter. Surgery revealed an intense metallosis resulting from contact of the dislocated expanding ring with the neck of the stem. Signs of rubbing and also polished spots on the titanium body of the neck were detected (Figures 2 and 3).

1 patient (case no. 6) had revision surgery due to stem loosening. The expanding ring was dislocated. The cup was revised too, changing the system to a Mueller antiluxation inlay. Shortly after this intervention, another dislocation occurred.

In 4 patients (cases no. 1, 3, 5, and 7) the follow-up examination revealed a luxation of the expanding ring. These patients reported no further dislocations and had no pain. However, they had limited mobility, which they regarded as an acceptable restriction to their quality of life considering that they had remained free of dislocations.

1 patient did not consent to a follow-up examination. At a phone interview, he expressed his dissatisfaction with the outcome because of subluxation symptoms and restrictions to movement. He did not mention any inpatient treatment or dislocations.

Radiographs from any of the cases did not reveal any signs of loosening of the Durolac cups or migration of the inlays regarding their fixation in the cup.

## Discussion

Our results are in line with the results of other studies, which have shown a high failure rate of the Duraloc Constrained Inlay (Della Valle et al. 2005, Williams et al. 2007). These publications focused on revision surgery as the main endpoint and did not present any specific information about patients without symptoms of implant failure. In our series, 4 of 8 patients treated with this product showed no clinical symptoms apart from restricted hip motion, but there was evidence of dislocation of the expanding ring—leading to revision surgery due to metallosis in 1 patient. The limited mobility after implantation may be explained by design-induced restrictions of motion within the implant.

The failure mechanism could conceivably be an impingement between the elevated edge of the inlay and the neck of the stem, which might finally squeeze out the expanding ring. In the Duraloc Constrained Inlay with a metal ring any damage of the elevated edge, which essentially accounts for the protective effect against dislocation, can be disclosed radiolographically. This does not always apply to implants made entirely of polyethylene; in such cases a normal radiograph cannot be regarded as a safe indicator of an undamaged edge of the implant and proper functioning.

It is noticeable that publications about implants with a similar basic philosophy as the Duraloc Constrained Inlay have also described implant failure, at least in individual cases (Kaper and Bernini 1998, Shapiro et al. 2003, Della Valle et al. 2005, Guyen et al 2007, Kapoor et al 2007, Williams et al. 2007). In the surgical therapy of recurrent dislocation, we now prefer the use of large head implants.
